# Enhancing Sky-Blue Perovskite Light-Emitting Diode Performance through Guanidinium-Based Dual-Functional Molecular Engineering

**DOI:** 10.1021/acsami.5c23193

**Published:** 2026-01-22

**Authors:** Yu-Hsiang Teng, Hou Li, Chiung-Han Chen, Yen-Yu Wang, Bi-Hsuan Lin, I-Chih Ni, Chi-Ching Kuo, Yu-Jung Lu, Chu-Chen Chueh

**Affiliations:** † Department of Chemical Engineering, 33561 National Taiwan University, Taipei 10617, Taiwan; ‡ Research Center for Applied Sciences, Academia Sinica, Taipei 11529, Taiwan; § National Synchrotron Radiation Research Center, Hsinchu 30076, Taiwan; ∥ Graduate Institute of Photonics and Optoelectronics, National Taiwan University, Taipei 10617, Taiwan; ⊥ Department of Molecular Science and Engineering, Institute of Organic and Polymeric Materials, National Taipei University of Technology, Taipei 106, Taiwan

**Keywords:** perovskite light-emitting diodes, sky-blue emission, dual-functional molecular engineering, defect passivation, phase distribution regulation

## Abstract

Perovskite light-emitting diodes (PeLEDs) have emerged as promising candidates for next-generation display and lighting technologies due to their high photoluminescence quantum yield, tunable emission characteristics, and narrow spectral bandwidth. However, achieving efficient and stable blue emission remains a significant challenge, primarily due to poor phase purity, excessive trap density, and unfavorable energy level alignment. Herein, we propose a dual-functional molecular engineering strategy utilizing 4-guanidinobenzoic acid hydrochloride (GBAC) as both a buried interfacial layer and a bulk additive. When employed at the buried interface, GBAC enhances surface wettability and precursor spreading, thereby improving film morphology and crystallinity. This additional layer also helps optimize energy level alignment between the hole transport layer and the perovskite emissive layer, reducing the injection potential barrier. Simultaneously, when acting as an additive in the bulk phase, GBAC’s guanidinium group binds to undercoordinated Pb^2+^ trap states via electrostatic coordination, suppressing nonradiative recombination; while its carboxylic group forms hydrogen bonds with the ammonium end of phenethylammonium bromide, reducing the formation of low-*n* (*n* = 1–2) phases and promoting growth in the medium-to-high n-value domains, thereby enhancing energy funneling efficiency. Leveraging these advantages, the device fabricated with dual GBAC treatment exhibits enhanced spectral stability, reduced turn-on voltage, and achieves an external quantum efficiency of up to 10.6% in the sky-blue emission band (∼489 nm), representing *a* > 60% improvement over the pristine device.

## Graphical abstract

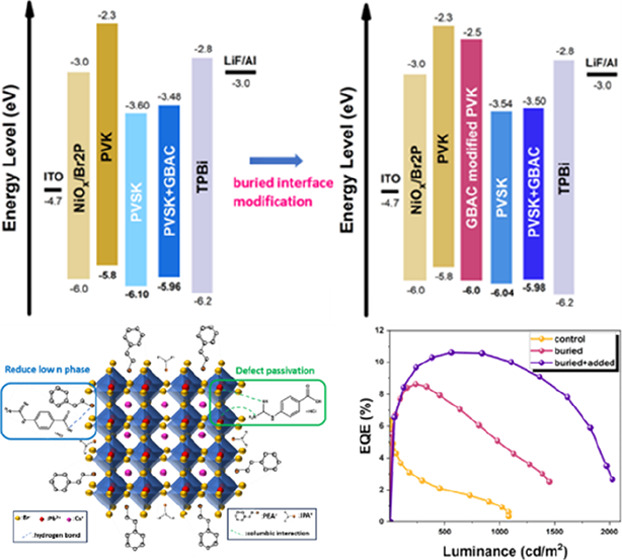

## Introduction

Light-emitting diodes (LEDs) have undergone rapid technological evolution. With their exceptional energy efficiency, long operating life, and outstanding switching capability, they have become indispensable components in modern lighting and display technologies.^1–4^ Among emerging candidate materials for next-generation LEDs, metal halide perovskites have garnered significant attention due to their solution-processability, tunable emission wavelengths, narrow emission line widths, and high photoluminescence quantum yield (PLQY).^5–8^ Over the past decade, perovskite LEDs (PeLEDs) have demonstrated significant progress, with their green and red emission external quantum efficiency (EQE) exceeding 30%.^9–11^ However, achieving highly efficient and stable blue PeLEDs remains an unresolved challenge, and this bottleneck severely limits the development of full-spectrum perovskite display technology.^12,13^

Blue perovskite emitters fact unique material and structural constraints. While all-bromide compositions are commonly employed to avoid phase separation prevalent in mixed-halide systems, they present inherent issues: high trap density, poor film uniformity, and suboptimal energy level alignment with adjacent charge transport layers.^14^ Furthermore, the quasi-2D perovskite structure, widely adopted to enhance exciton confinement and luminescence efficiency, requires a delicate balance among multiple characteristics: low-*n* phases (*n* = 1–3), while offering suitable blue emission bandgaps, often suffer from suppressed charge mobility and enhanced exciton–phonon interactions, leading to nonradiative losses and spectral instability.^15,16^

To effectively overcome these challenges, strategies that simultaneously modulate crystallization behavior, optimize phase distribution, and control energy level alignment are crucial. For example, Sheng et al. employed the bifunctional ligand 2-amino-1,3-propanediol (APDO) to guide crystal growth in the quasi-2D film.^17^ Meng et al. exploited triethylammonium chloride (TEAC) to narrow the phase distribution within the quasi-2D film.^18^ Zhang et al. introduced a bis-4-(*N*-carbazolyl)­phenyl)­phenylphosphine oxide (BCPO) interlayer between PEDOT/PSS and the perovskite layer, enhancing film smoothness and hole transport.^19^ These research findings highlight the pivotal role of molecular interfaces and additive engineering in overcoming the longstanding limitations of blue perovskite luminescent materials.

In this study, we propose a dual-functional molecular engineering approach using 4-guanidinobenzoic acid hydrochloride (GBAC), which simultaneously functions as a buried interface modulator and an internal additive within the perovskite light-emitting layer. GBAC possesses both guanidino and carboxyl groups, endowing it with multisite interaction capabilities. As a buried interface modifier, GBAC improves the wettability and surface energy of the underlying hole transport layer, promoting uniform nucleation and growth of perovskite films. This enhances crystal orientation, reduces interfacial defects, thereby boosting hole injection efficiency and minimizing interfacial recombination. Simultaneously, when serving as an internal additive, GBAC interacts with undercoordinated Pb^2+^ and organic cations (e.g., PEABr) via hydrogen bonds and electrostatic interactions. These interactions passivate defect sites and regulate crystallization kinetics, enabling perovskite films to achieve higher phase purity, suppress low-*n* domains, and enhance PLQY. Ultimately, the optimized PeLED demonstrates significantly enhanced spectral stability and EQE in the sky-blue region, marking a promising engineering strategy toward high-performance blue PeLEDs.

## Experimental Section

### Materials

Nickel­(II) acetate tetrahydrate [Ni­(CH_3_COO)_2_·4H_2_O] was purchased from Alfa Aesar. Ethanol was purchase from Echo Chemcial Co. Ltd. Lead bromide (PbBr_2_, >99.9%), (2-(3,6-dibromo-9*H*-carbazol-9-yl)­ethyl)­phosphonic acid (Br-2PACz, >99%), 4-guanidinobenzoic acid hydrochloride (GBAC) were purchased from Tokyo Chemical Industry Co., Ltd. Ethanolamine (NH_2_CH_2_CH_2_OH, 99.5%), 2-propanol­(anhydrous, 99.5%), poly­(9-vinylcarbazole) (PVK, number-average molecular weight (*M*_n_) range from 25,000 to 50,000 g mol^–1^), chlorobenzene (anhydrous, 99.8%), ethyl acetate (anhydrous, 99.8%), phenethylammonium bromide (PEABr, ≥98%), cesium bromide (CsBr, >99.9%), iso-propylammonium bromide (IPABr, 99.5%), lithium fluoride (LiF, >99.99%), and the solvents dimethyl sulfoxide (DMSO, ≥99.9%) and dimethyl sulfoxide-d6 (DMSO-*d*_6_, ≥99.5%) were purchased from Sigma-Aldrich. 2,2′,2″-(1,3,5-benzinetriyl)-tris­(1-phenyl 1-Hbenzimidazole) (TPBi) was purchased from Ultra Fine Chemical Technology Corp. All commercial materials were used directly without further purifications.

### Preparation of Precursor Solutions

The solution formulation for quasi-2D sky-blue perovskite is as follows: dissolve 38.4 mg of CsBr, 110.2 mg of PbBr_2_, 48.4 mg of PEABr, and 16.8 mg of IPABr in 2 mL of DMSO. For films requiring molecular doping, GBAC was introduced into the precursor solution at a concentration of 0.81 mg/mL. The hole transport layer (PVK) solution was prepared by dissolving 6 mg of PVK in 1 mL of chlorobenzene. The self-assembled monolayer (Br-2PACz) solution was prepared by dissolving 3 mg of Br-2PACz in 4 mL of 2-propanol. For the GBAC surface modification layer, 1 mg of GBAC was dissolved in 1 mL of 2-propanol. All aforementioned solutions were prepared in a nitrogen-filled glovebox under ambient conditions with magnetic stirring overnight to ensure complete dissolution and homogenization. The preparation method for the nickel oxide (NiO_*x*_) precursor solution is as follows: dissolve nickel­(II) acetate tetrahydrate (Ni­(CH_3_COO)_2_·4H_2_O) and ethanolamine (NH_2_CH_2_CH_2_OH) in anhydrous ethanol, maintaining a molar ratio of metal salt to complexing agent of 1:1. The total Ni^2+^concentration is adjusted to 0.2 M. The mixture was stirred continuously overnight at 70 °C in a glovebox prior to use.

### Device Fabrication

Indium–tin-oxide (ITO) glass substrates were first washed with laboratory detergent, followed by sequential ultrasonic cleaning in deionized water, acetone and 2-propanol for 20 min each. After drying with N_2_, the substrates were placed in a 60 °C oven to remove residual solvents. Subsequently, an 8 min air-plasma treatment was performed. For the hole-injection layer, a NiO_*x*_ precursor filtered through a 0.45 μm PTFE filter was spin-cast at 3000 rpm for 50 s. This was followed by thermal annealing at 270 °C for 45 min under ambient conditions. After cooling the films to room temperature, they underwent 2 min of UV–ozone exposure to enhance surface wettability. The samples were transferred to a N_2_-purged glovebox. A self-assembled monolayer (Br-2PACz) was spin-coated at 2000 rpm for 30 s and cured at 100 °C for 10 min. Subsequently, PVK was coated under identical spin-coating conditions and baked at 150 °C for 30 min. Next, the GBAC solution was spin-coated at 5000 rpm for 30 s and briefly baked at 100 °C for 1 min to introduce GBAC as an interfacial modifier. The quasi-2D sky-blue perovskite precursor was spin-coated at 4000 rpm for 120 s; followed by a 30 s drop of 100 μL ethyl acetate as an antisolvent. Films were crystallized by heating at 70 °C for 10 min. The electron transport layer and top electrodes were thermally evaporated (base pressure ∼ 1 × 10^–6^ Torr): TPBi (30 nm), LiF (1 nm) and Al (100 nm). A 0.10 cm^2^ effective pixel area was defined using a shadow mask. All wet-processing steps following UV–ozone treatment were performed in a N_2_ glovebox. For space-charge-limited-current (SCLC) analysis, hole-only devices with a architecture of ITO/NiO_*x*_/Br-2PACz/PVK/GBAC/perovskite (with or without bulk GBAC)/MoO_3_/Ag were fabricated. The MoO_3_ (8 nm) and Ag (100 nm) layers were deposited by thermal evaporation under the same high-vacuum conditions as described above.

### Characterization

The optical absorption characteristics of the samples were evaluated using a UV–visible spectrophotometer (Hitachi U-4100). Static contact angle measurements were performed with a goniometric contact angle system to assess surface wettability. Optical bandgap values for the samples were estimated from Tauc plots derived from UV–vis absorption spectra. To investigate the crystallographic characteristics, X-ray diffraction (XRD) measurements were performed using a Rigaku SmartLab SE diffractometer. The surface morphology and microstructure of the perovskite films were observed in high-resolution mode using a field-emission scanning electron microscope (FE-SEM, Hitachi S-4800). Photoluminescence (PL) and time-resolved photoluminescence (TRPL) analyses were conducted at the Taiwan photon source (TPS) 23A beamline at the National Synchrotron Radiation Research Center (NSRRC, Taiwan). Measurements were performed using a HORIBA iHR320 spectrometer equipped with a Hamamatsu C10910 streak camera and M10913 slow-scan module to enhance temporal resolution. Room-temperature electrochemical impedance spectroscopy (EIS) testing of encapsulated devices was performed using a BioLogic SP-200 system, applying AC perturbations across a wide frequency range. Capacitance–voltage (*C*–*V*) measurements were executed via Fluxim AG’s the Paios platform to investigate device charge storage behavior. To fully characterize the photoluminescent properties of PeLEDs, including current density–voltage–luminance (*J–V–L*), external quantum efficiency (EQE), and electroluminescence (EL) spectra, testing was performed using a LQ-100 test station (Enlitech Co., Ltd.) integrated with a 100 mm diameter integrating sphere and a source-measure unit (B2901A, Keysight Technologies). Elemental and chemical-state analysis was performed using an X-ray photoelectron spectroscopy (XPS, PerkinElmer PHI 5400) equipped with a monochromated Al Kα source. Molecular vibration and functional group analysis was obtained using a Fourier-transform infrared spectroscopy (FTIR, PerkinElmer Spectrum Two L16000), while nuclear magnetic resonance (NMR) spectra were recorded using a Bruker DPX 400 MHz spectrometer.

## Result and Discussion

To investigate the impact of dual-functional molecular engineering on blue PeLEDs, we herein employed GBAC as both a buried interface modifier and an internal additive with the perovskite layer. The preparation process is illustrated in Figure 1a: First, a GBAC solution in IPA was spin-coated onto the hole transport layer (HTL). Following thermal annealing, a uniform GBAC-modified interfacial layer was formed. The guanidinium cations and carboxylic acid functional groups in GBAC create multiple interaction sites with the perovskite precursor, enhancing interface compatibility and improving the quality of the prepared perovskite film. Subsequently, GBAC was incorporated as an additive into the perovskite matrix, and a quasi-2D perovskite luminescent layer was fabricated on the GBAC-modified HTL. Functional groups within GBAC interact with Pb^2+^ and ammonium-based A-site cations (e.g., PEABr). Such interactions can passivate undercoordinated Pb sites, regulate nucleation and growth processes, thereby enhancing crystal quality and suppressing low-*n* phase formation.^20–22^ The figure also depicts GBAC’s molecular structure and its dual interaction modes—forming hydrogen bonds with NH_3_^+^ and coordinating with Pb^2+^—accompanied by a schematic of the quasi-2D perovskite lattice. The ultimate goal is to efficiently control phase distribution and enhance crystalline quality, thereby realizing high-performance sky-blue PeLEDs.

**Figure 1:**
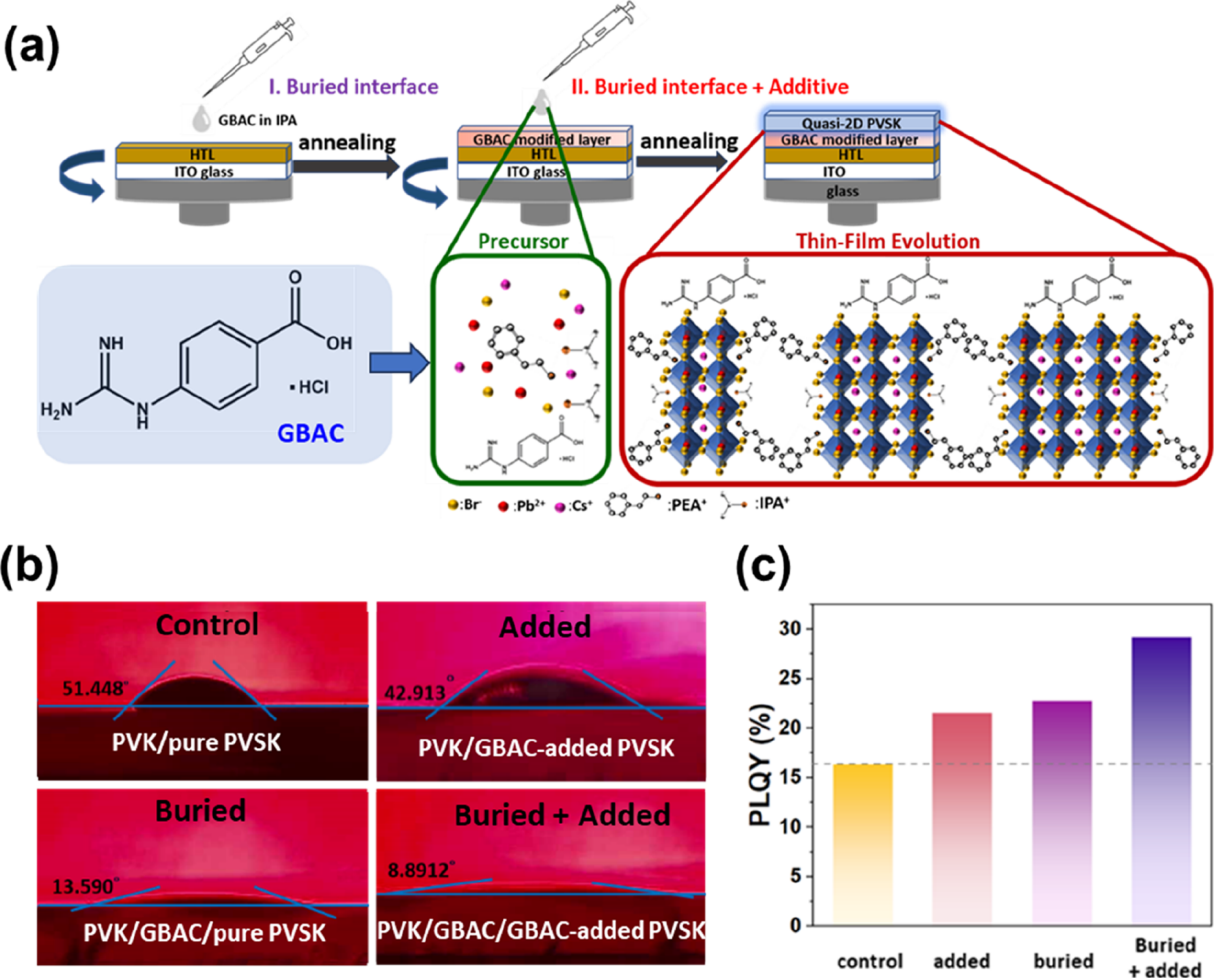
(a) Schematic of the device preparation process and the dual-functional role of GBAC in the buried interface and additive engineering. (b) Contact angle measurements of perovskite films with and without GBAC modification at the buried interface and/or bulk phase. (c) PLQY chart of control and different GBAC-treated perovskite films.

We first examined the wettability of different substrate-precursor combinations via contact angle measurements (Figure 1b). The control sample (composed of a pristine PVK layer and unmodified perovskite, i.e., PVK/pure PVSK) exhibited a relatively large contact angle (51.448°), indicating poor wettability and restricted precursor spreading. When GBAC was added to the perovskite (PVK/GBAC-added PVSK), the contact angle decreased to 42.913°. This change is clearly attributed to the presence of polar GBAC molecules. A more pronounced effect was observed when the underlying PVK was modified with a GBAC buried interlayer. The PVK/GBAC/pure PVSK sample exhibited a contact angle of 13.590°, reflecting significantly enhanced interfacial compatibility. When GBAC was present both at the buried interface and in the bulk phase (PVK/GBAC/GBAC-added PVSK), the contact angle further decreased to 8.891°, indicating near-complete wetting. This optimized wetting behavior is a key factor in achieving uniform crystallization and compact film morphology.^23–26^ This result highlights GBAC’s dual role: at the interface, its guanidino and carboxylic groups enhance surface polarity, promoting uniform precursor distribution during spin coating; simultaneously, GBAC incorporated within the perovskite layer modifies precursor viscosity and coordination environment, further facilitating uniform nucleation.

We then evaluated the photophysical properties of perovskite films under different GBAC treatment conditions. As shown in Figure 1c, the PLQY of the control film was 16.4%. Upon introducing GBAC as an additive or as a buried interfacial layer, the PLQY increased to 21.6% and 22.8%, respectively. Remarkably, when GBAC was simultaneously incorporated at both the interface and within the perovskite (buried + added), the PLQY significantly increased to 29.2%. This substantial improvement indicates that GBAC effectively passivates trap states and enhances radiative recombination efficiency. Subsequent sections will focus on comparing three representative conditions—control, buried interface, and buried + added—through detailed morphological, structural, and optoelectronic characterization.

We further performed scanning electron microscopy (SEM) measurements on films prepared under these three conditions. As shown in Figure 2a, the pristine quasi-2D perovskite exhibits a relatively indistinct surface morphology with poorly defined grain boundaries. The overall surface appears rough and discontinuous, featuring voids or microcracks. Notably, this SEM appearance does not imply an amorphous crystal structure; XRD/wide-angle X-ray scattering (GIWAXS) still reveals distinct diffraction peaks, indicating the presence of crystalline quasi-2D domains. Upon introducing GBAC as a buried interlayer, the perovskite film displays more pronounced rod-like or flake-like crystalline grains, with improved domain orientation and alignment (Figure 2b). This observation indicates that the GBAC interlayer promotes oriented crystal growth and enhances nucleation density, thereby improving film crystallinity and coverage.^23–26^ The film with dual GBAC treatment (buried + added) reveals the densest and most clearly defined morphology (Figure 2c). Grain shapes resemble those in the buried interlayer case but feature higher intergranular connectivity and a more uniform overall film distribution. These features collectively reflect an optimized crystallization process: GBAC not only promotes interface-oriented nucleation but also modulates bulk crystal growth dynamics, thereby achieving seamless grain fusion and suppressing phase impurities.

**Figure 2:**
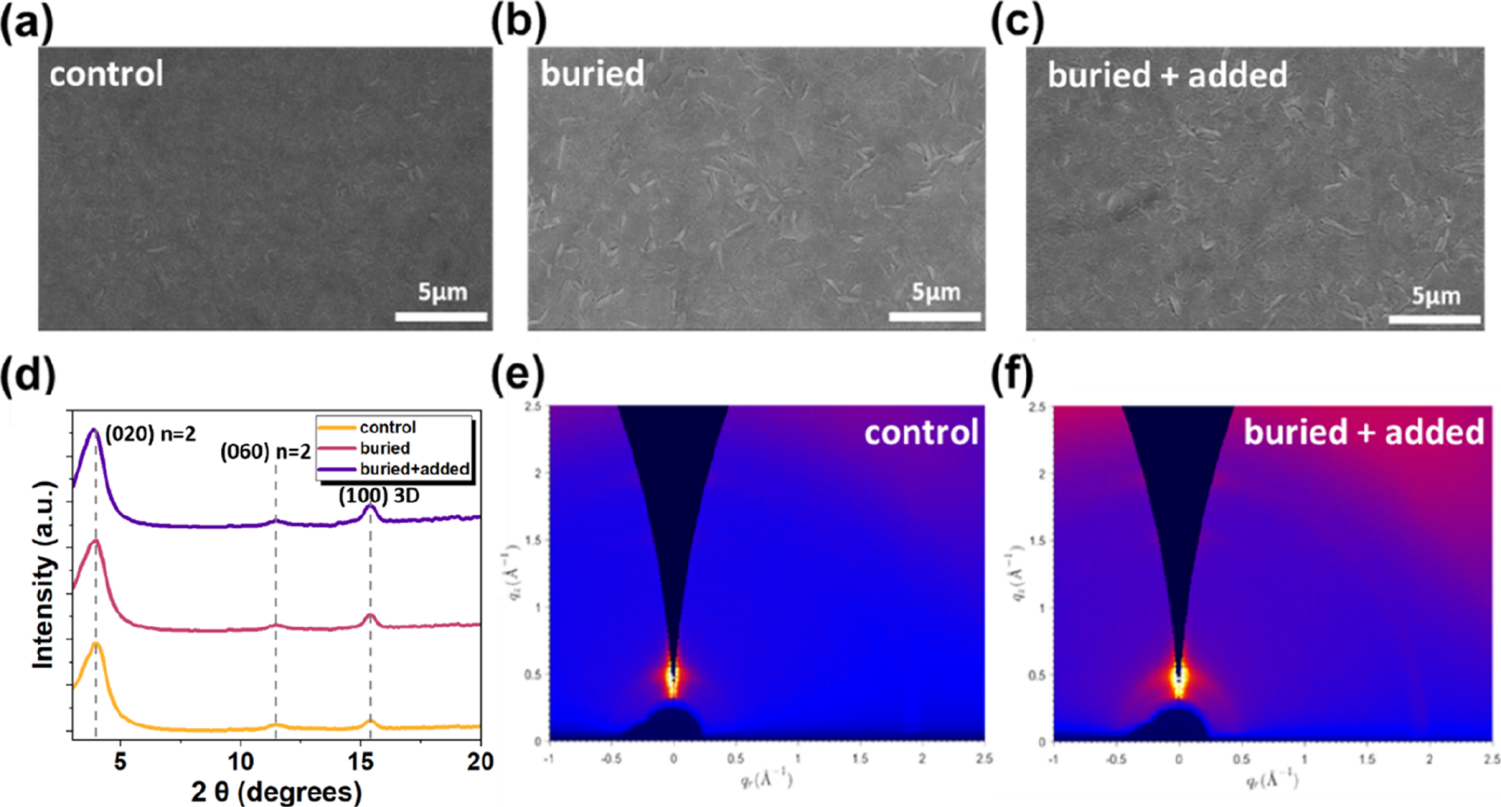
SEM images of (a) control, (b) buried, and (c) buried + added perovskite films. (d) XRD patterns of these perovskite films. GIWAXS patterns of (e) control and (f) buried + added perovskite films.

However, the origin of the prominent flake-like features observed in SEM images remains unclear. To further elucidate the nature of these structures and their correlation with film crystallinity, we subsequently performed XRD measurements for a more comprehensive structural analysis. As shown in Figure 2d, all samples exhibited a distinct diffraction peak at 2θ ≈ 3.93°, corresponding to the (020) {*n* = 2} reflection plane of the quasi-2D perovskite phase.^27–29^ Notably, the intensity of this peak remained comparable across the control, buried, and buried + added samples, indicating that the formation of the *n* = 2 layered structure was maintained regardless of GBAC treatment. It should be noted that the θ–2θ XRD intensity is largely on orientation average; consequently, subtle variations in the thin-film texture and vertical stacking coherence of layered quasi-2D domains may not manifest as pronounced intensity changes in the θ–2θ scan. In contrast, more significant intensity changes are observed at 2θ ≈ 15.4° (corresponding to the (100) crystal plane of the 3D-like perovskite phase).^27,29^ The control film exhibited a relatively weak and broad (100) peak, indicating poor crystallinity and limited long-range order. Upon introducing GBAC at the buried interface, the diffraction peak became markedly sharper and more intense, suggesting improved nucleation and grain growth. The strongest and narrowest (100) diffraction signal was obtained under the buried + added condition, indicating enhanced development of the 3D phase, improved grain orientation, and reduced structural disorder. These results demonstrate that GBAC incorporation promotes the growth of 3D-like domains and enhances the overall crystallinity of the film.

To further understand the crystal orientation and phase organization of perovskite films, grazing incidence GIWAXS measurements were subsequently performed. As shown in 2D GIWAXS patterns (Figures 2e,f and S1), the control sample exhibited relatively isotropic diffraction rings, particularly centered at *q* ≈ 0.5 Å^–1^. This corresponds to the (020) plane of the *n* = 2 phase.^30,31^ This isotropy indicates the presence of disordered microstructure and random crystal orientation, consistent with the blurred grain boundaries and discontinuous surface morphology observed in SEM images, as well as the broad diffraction peaks in the XRD pattern—particularly the weak (100) peak at 2θ ≈ 15.4°. Upon introducing GBAC as a buried interfacial layer, the GIWAXS patterns revealed a notable increase in diffraction intensities both in-plane and out-of-plane directions. This indicates not only improved vertical orientation of quasi-2D layers (evident by stronger out-of-plane scattering) but also superior lateral crystallinity and interphase connectivity (supported by enhanced in-plane scattering at *q*_*x*_ > 1.5 Å^–1^). The buried + added sample exhibited the most pronounced intensities in both in-plane and out-of-plane scattering, reflecting the synergistic effect of GBAC in promoting highly ordered stacking and grain growth. This conclusion is further validated by in-plane and out-of-plane linecuts (Figure S2). In the out-of-plane profiles, the intense diffraction peak at *q*_*z*_ ≈ 0.5 Å^–1^ corresponds to the (020) plane of the *n* = 2 quasi-2D perovskite phase. The intensity of the (020) peak progressively increases from the control sample to the buried sample and then to the buried + added sample, indicating improved preferred orientation and vertical stacking coherence within the quasi-2D layered domains. Notably, this enhanced (020) peak intensity in GIWAXS linecuts primarily reflects improved orientation and vertical stacking coherence rather than a direct linear increase in the absolute *n* = 2 phase fraction. Therefore, the evolution of the *n*-phase fraction was investigated based on the UV–vis deconvoluted phase histogram (Figure 3b). Additionally, high-*q* reflection peaks (*q*_*z*_ ≈ 1.5 Å^–1^ and 2.0 Å^–1^) exhibited notable intensity growth. These peaks correspond to the (100) and (110) crystal planes of 3D-like phases, reflecting improved crystal structure and the formation of 3D-like domains. Similar trends were observed in the in-plane linecuts. All samples exhibited multiple diffraction peaks corresponding to quasi-2D perovskite phases with varying n values. The buried + added sample demonstrated the highest intensity across these peaks, indicating more pronounced in-plane order and enhanced phase purity. These results demonstrates that GBAC treatment promotes more uniform and compact crystallization while improving vertical charge transport pathways and lateral grain connectivity. This conclusion is consistent with aforementioned SEM images and XRD data.

**Figure 3:**
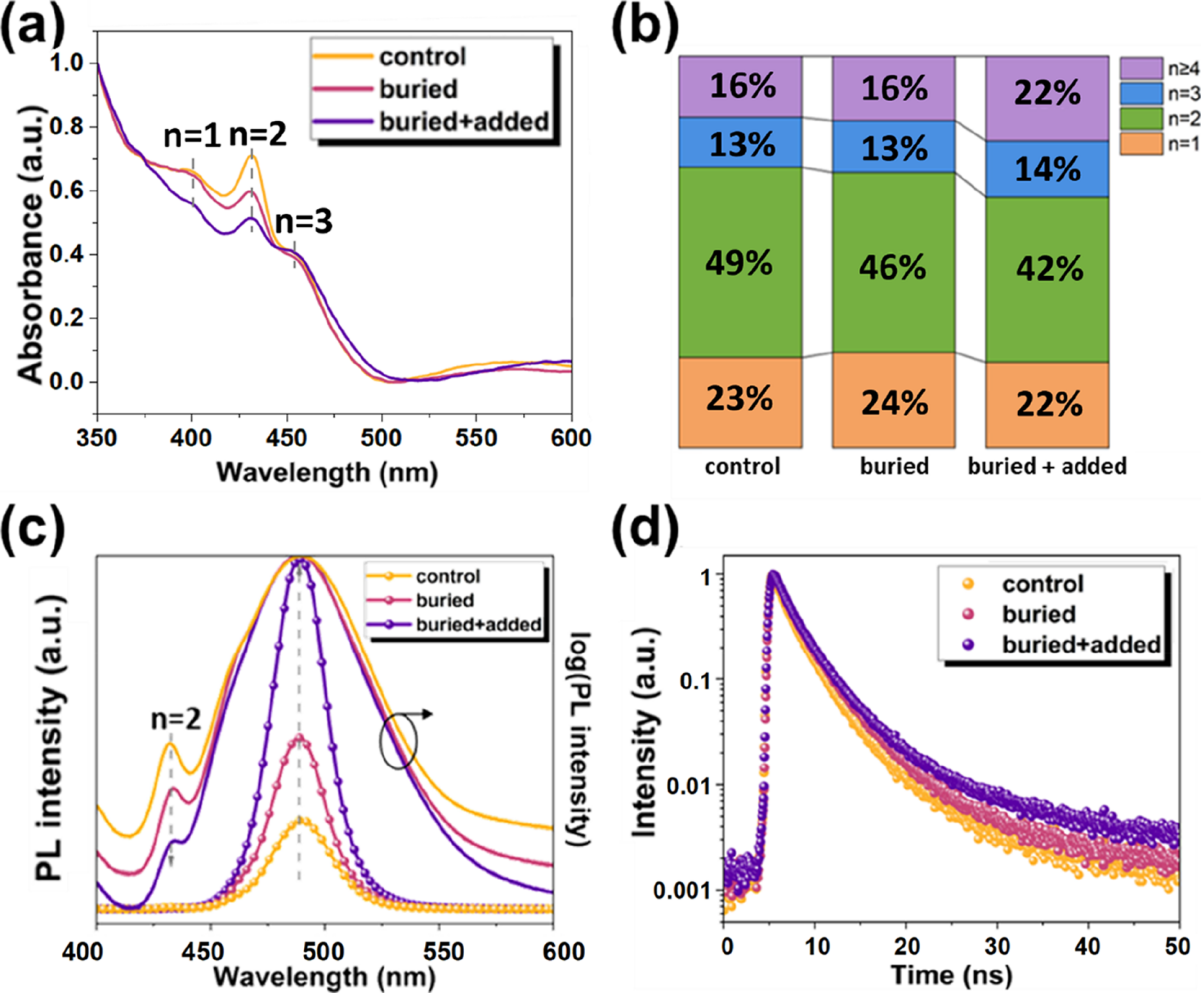
(a) UV–vis absorption spectra of perovskite films with and without different GBAC treatments. (b) Corresponding phase composition histograms. (c) Steady-state PL spectra of perovskite films with and without different GBAC treatments and their corresponding logarithmic-scale representations. (d) Time-resolved PL spectra of perovskite films with and without different GBAC treatments.

We further analyzed the phase distribution of quasi-2D perovskite films via UV–vis absorption spectroscopy, with results shown in Figure 3a. The absorption spectra reveal the relative distribution of different n phases based on their characteristic exciton peaks. The control film exhibits distinct absorption peaks at ∼400 nm (*n* = 1) and 430 nm (*n* = 2), indicating a significant low n-phase content.^28,32^ Although these phases demonstrate strong exciton confinement effects, their low carrier mobility and high trap density typically hinder charge transport. In contrast, the low-*n* phase absorption peaks in the buried + added film are significantly attenuated, with a pronounced shoulder peak appearing in the 460–500 nm range corresponding to quasi-2D phases (*n* ≥ 4). The distinct absorption peak observed around 460 nm can be attributed to the *n* = 3 phase.^28^ These spectral changes indicate that GBAC incorporation, whether at the buried interface or as an additive, not only suppresses the formation of low-dimensional domains (*n* = 1–2) but also promotes the growth of medium-to-high n phases. To further quantify phase distribution, Figure 3b presents a Gaussian-deconvoluted histogram of the absorption spectra, where the integrated area under each fitted peak estimates the relative content of different n phases. As shown, under the buried + added condition, the proportions of *n* = 1 and 2 phases are significantly reduced, while the contributions from *n* = 3 and *n* ≥ 4 phases increase. This compositional evolution indicates a more optimized phase organization and smoother energy gradient (energy cascade) between *n*-domains. In mixed *n*-domain quasi-2D perovskite films, this energy landscape is typically described by the multiple-quantum-well (MQW) energy-cascade model: excitation generated in the wider-bandgap low-*n* domains can be transferred downward to the narrower-bandgap high-*n* (quasi-3D) luminescent convergence domains (exciton/energy funneling effect). Therefore, by regulating the *n*-phase distribution and interdomain coupling, the energy transfer pathway can be optimized, reducing loss during interdomain relaxation processes. This promotes radiative recombination and enhances luminescence efficiency.^33^

Figure 3c presents the steady-state PL spectra of the control, buried, and buried + added perovskite films, with the left *Y*-axis plotted on a linear intensity scale (symbols) and the right *Y*-axis displayed in logarithmic scale (solid lines), both axes corresponding to the same data set. All samples exhibit a dominant emission peak around 489 nm, confirming their common emissive origin. Compared to the control sample, the buried and buried + added films demonstrate significantly higher PL intensity, consistent with their enhanced PLQY. Examining the log-scale curves reveals a subtle shoulder peak around 430 nm in the control film, attributable to a low-dimensional phase (*n* = 2). This feature is strongly suppressed after GBAC modification, indicating reduced low-*n* domains. The trend correlates with UV–vis absorption results. Suppressing the low-*n* emission implies reduced exciton localization, thereby enabling more efficient exciton/energy funneling along energy cascade pathways toward high-*n* emission convergence regions, where radiative recombination predominantly occurs. Notably, despite an increased contribution from midto-high-*n* domains after GBAC treatment, the primary PL peak remains centered around ∼489 nm without significant red shift. This occurs because the peak position is primarily determined by the characteristics of the dominant emissive domains, while phase redistribution is more sensitively reflected in the suppression of shoulder peaks in the low-*n* phases and changes in intensity/line width. Furthermore, the energy gaps between adjacent midto-high-*n* domains may be relatively small compared to spectral broadening and reabsorption effects—phenomena that could mask minor peak position shifts.^33^

Time-resolved PL (TRPL) measurements were then performed to probe carrier recombination kinetics, as shown in Figure 3d. The biexponential decay fitting results (detailed in Table S1) reveal that the average lifetime progressively increased from 2.40 ns (control) to 2.68 ns (buried), and was further extended to 2.87 ns under the buried + added condition. This lifetime enhancement indicates that the effective trap passivation suppresses nonradiative recombination.^34,35^ To further elucidate the mechanism behind the enhanced average carrier lifetime, we performed a correlation analysis between the TRPL data and the PLQY results. By definition, PLQY quantifies the proportion of radiative recombination relative to total recombination processes, expressed as the ratio of *k*_rad_ to *k*_rad_ + *k*_nonrad_, where *k*_rad_ and *k*_nonrad_ represent radiative and nonradiative recombination rates, respectively. Additionally, the average carrier lifetime can be described as the reciprocal of the total recombination rate, i.e., τ_avg_ = 1/(*k*_rad_ + *k*_nonrad_).^36,37^ Based on these relationships, we extracted the individual values of *k*_rad_ and *k*_nonrad_ and complied them in Table S1. The results show that the radiative recombination rate constant (*k*_rad_) increased from 6.82 × 10^7^ s^–1^ (control) to 10.2 × 10^7^ s^–1^ (buried + added), while the nonradiative recombination rate constant (*k*_nonrad_) decreased correspondingly. This trend aligns with the observed results for the PLQY and steady-state PL.

To understand the molecular-level interactions between GBAC and perovskite components, we conducted detailed spectroscopic analyses including nuclear magnetic resonance (NMR), Fourier-transform infrared spectroscopy (FTIR), and X-ray photoelectron spectroscopy (XPS). Here, solution-state ^1^H NMR and powder FTIR are employed as model systems to investigate the intrinsic chemical affinity of GBAC functional groups (guanidinium/–COOH) toward perovskite-related species and to identify potential interaction modes (e.g., ionic interactions and hydrogen bonding). We note that these ex situ measurements cannot provide a fully corresponding structural fingerprint of the final film configuration, as the local environment in spin-coated films is heterogeneous and rapidly evolves during solvent evaporation and crystallization (e.g., effective concentration/ionic strength, solvation, and interfacial constraints). Therefore, the NMR/FTIR results are interpreted as qualitative evidence of interaction tendencies, while film relevance is validated through film-based characterization measured directly on perovskite films (XPS binding-energy trends, GIWAXS/XRD evolution, and optical/dynamic features. First, ^1^H NMR spectroscopy was employed to probe the interactions between GBAC and the perovskite precursors in solution. As shown in Figure 4a, upon adding PbBr_2_, the NH proton signals (peaks 5 and 6) of the guanidinium group in GBAC shifted from 7.76 to 7.58 ppm in the low-field direction, while peak 4 also shifted from 10.40 to 9.88 ppm. This indicates a weakening of the shielding effect due to coordination with Pb^2+^ ions, confirming electrostatic interactions between the electron-rich guanidinium nitrogen and the undercoordinated Pb^2+^ ions.^38^ In the GBAC + PEABr system, the aromatic proton of PEABr (peak 7) exhibited a shift from 7.89 to 8.08 ppm. Concurrently, the COOH proton (peak 1) of GBAC exhibited peak broadening, suggesting hydrogen bonding formation between the GBAC carboxyl group and the ammonium end of PEABr. These interactions are crucial for regulating the crystallization process and suppressing excessive growth of low-*n* phase.^29^

**Figure 4:**
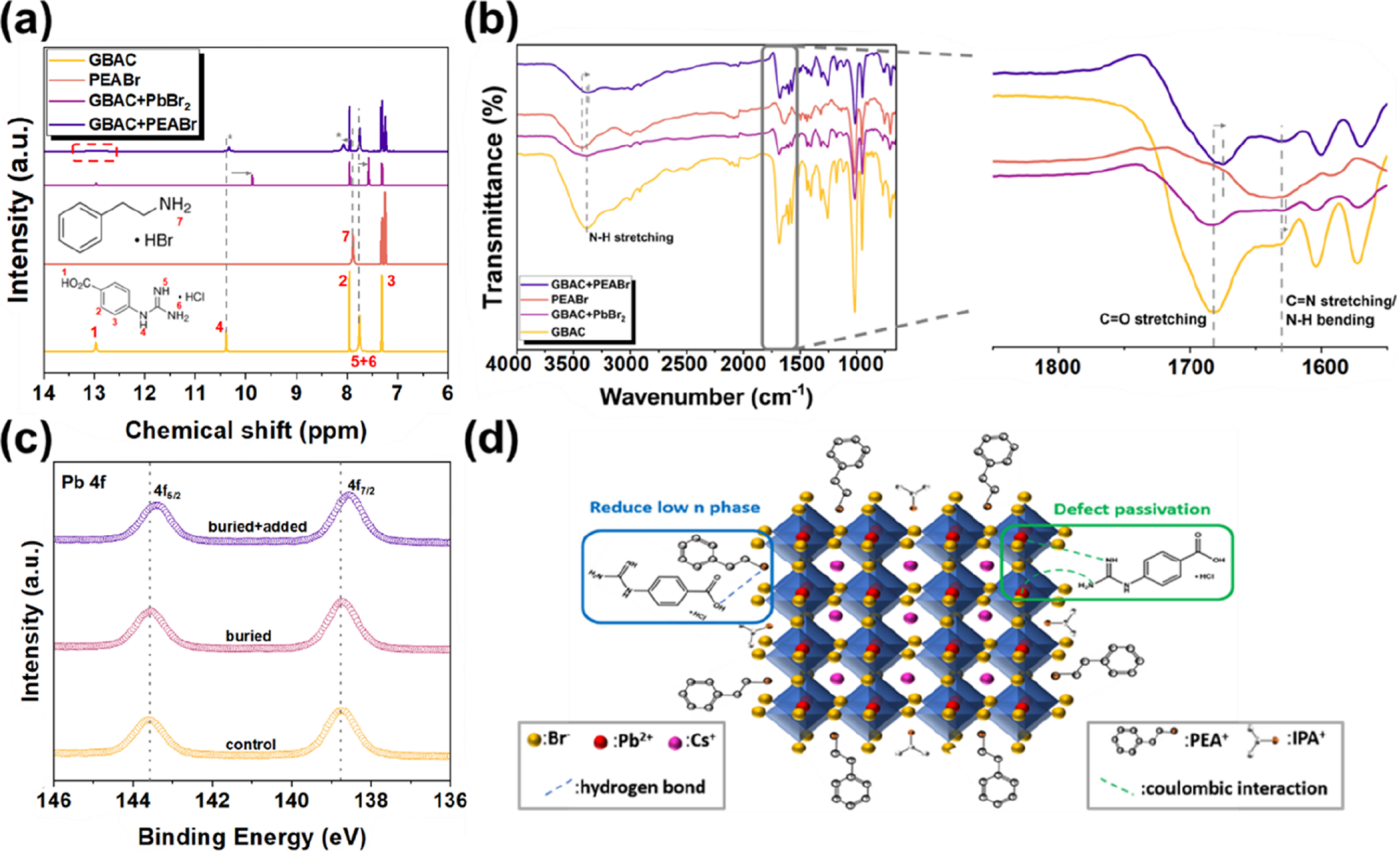
(a) ^1^H NMR spectra of GBAC, PEABr, GBAC + PbBr_2_, and GBAC + PEABr solutions. (b) FTIR spectra of GBAC, PEABr, GBAC + PbBr_2_, and GBAC + PEABr samples and the magnified FTIR spectrum in the 1600–1800 cm^–1^ range. (c) Pb 4f core-level XPS spectra for control, buried, and buried + added conditions. (d) Schematic illustration of the dual-functional role of GBAC in quasi-2D perovskite films.

FTIR spectroscopy further corroborates these findings. As showed in Figure 4b, when GBAC was mixed with PbBr_2_, the C=N stretching vibration and N–H bending vibration peaks shifted from 1630.2 cm^–1^ to 1626.8 cm^–1^, confirming the interaction between the guanidinium group and Pb^2+^ ion via electrostatic attraction. In the GBAC + PEABr mixture, the C=O stretching peak shifted from 1681.8 to 1674.5 cm^–1^, while the N–H stretching vibration peaks of PEA and GBAC shifted from 3382.8/3427.2 cm^–1^ to 3367.6 cm^–1^, respectively. These shifts confirm hydrogen bonding formation between GBAC’s carboxyl group and the terminal NH_3_^+^ group of PEABr.^39^ XPS analysis provides direct evidence of electronic interactions between GBAC and Pb^2+^ ions within films. As showed in Figure 4c, the Pb 4f peaks of the control sample were located at 143.61 eV (4f_7/2_) and 138.75 eV (4f_5/2_), consistent with the characteristic binding energies of Pb^2+^ in quasi-2D perovskites. When GBAC is introduced as a buried interlayer, only a slight shift in Pb 4f peaks was observed, indicating minor perturbation of the Pb coordination environment. However, when GBAC was applied simultaneously at the buried interface and as bulk additives (buried + added), the Pb 4f peaks shifted to 143.42 eV (4f_7/2_) and 138.57 eV (4f_5/2_). This distinct shift suggests reduced electron density around the Pb^2+^ center, attributable to electrostatic interactions between the electron-deficient guanidinium group in GBAC and the Pb^2+^ ion.^38^ These results indicate that the dual-introduced GBAC modulates the local chemical environment of Pb, potentially passivating undercoordinated sites and suppressing trap-assisted charge recombination. All binding energies were charge-corrected by referencing the incidental C 1s peak to 284.8 eV. Under our acquisition and fitting conditions, the uncertainty in the extracted peak positions was estimated to be within ±0.05 eV. Therefore, despite the small observed displacement magnitude (∼0.1–0.2 eV), it remains significant relative to the experimental uncertainty. These shits are regarded as supporting evidence for GBAC-induced alterations in the local chemical environment (e.g., changes in Pb–Br coordination/electrostatic interactions and associated defect passivation), rather than serving as independent proof. Similar trends were observed in the Br 3d and N 1s XPS spectra (Figure S3). Figure 4d schematically summarizes these dual interactions. The guanidinium group of GBAC interacts with Pb^2+^ ions via electrostatic forces, passivating traps and prolonging carrier lifetimes; simultaneously, its carboxylic group forms hydrogen bonds with PEABr, thereby regulating the crystallization process and suppressing the formation of low-*n* quasi-2D phases.

To elucidate the carrier transfer dynamics and phase evolution in quasi-2D perovskite films under different GBAC treatments, transient absorption spectroscopy (TAS) was employed for analysis. Figure 5a–c displays the 2D TAS contour maps of perovskite films with different GBAC treatments. Distinct ground-state bleaching (GSB) peaks at ∼400, 430, 460, and 480 nm correspond to the *n* = 1, 2, 3, and *n* ≥ 4 phases, respectively, consistent with UV–vis absorption results. Compared to the control, the buried + added film exhibits stronger GSB signals in the long-wavelength range (*n* ≥ 3) and suppressed low-*n* components, indicating a phase distribution shift toward higher-*n* domains. Figure 5d–f displays the TAS decay evolution over a delay range from 0.02 to 500 ps. In all cases, the high-energy GSB signals decay rapidly, while the long-wavelength signals persist, indicating sequential energy transfer from low-*n* to high-*n* phases. Notably, the buried + added film exhibits more uniform spectral evolution and prolonged retention of the high-*n* signal, suggesting enhanced phase coupling and suppressed charge recombination.

**Figure 5:**
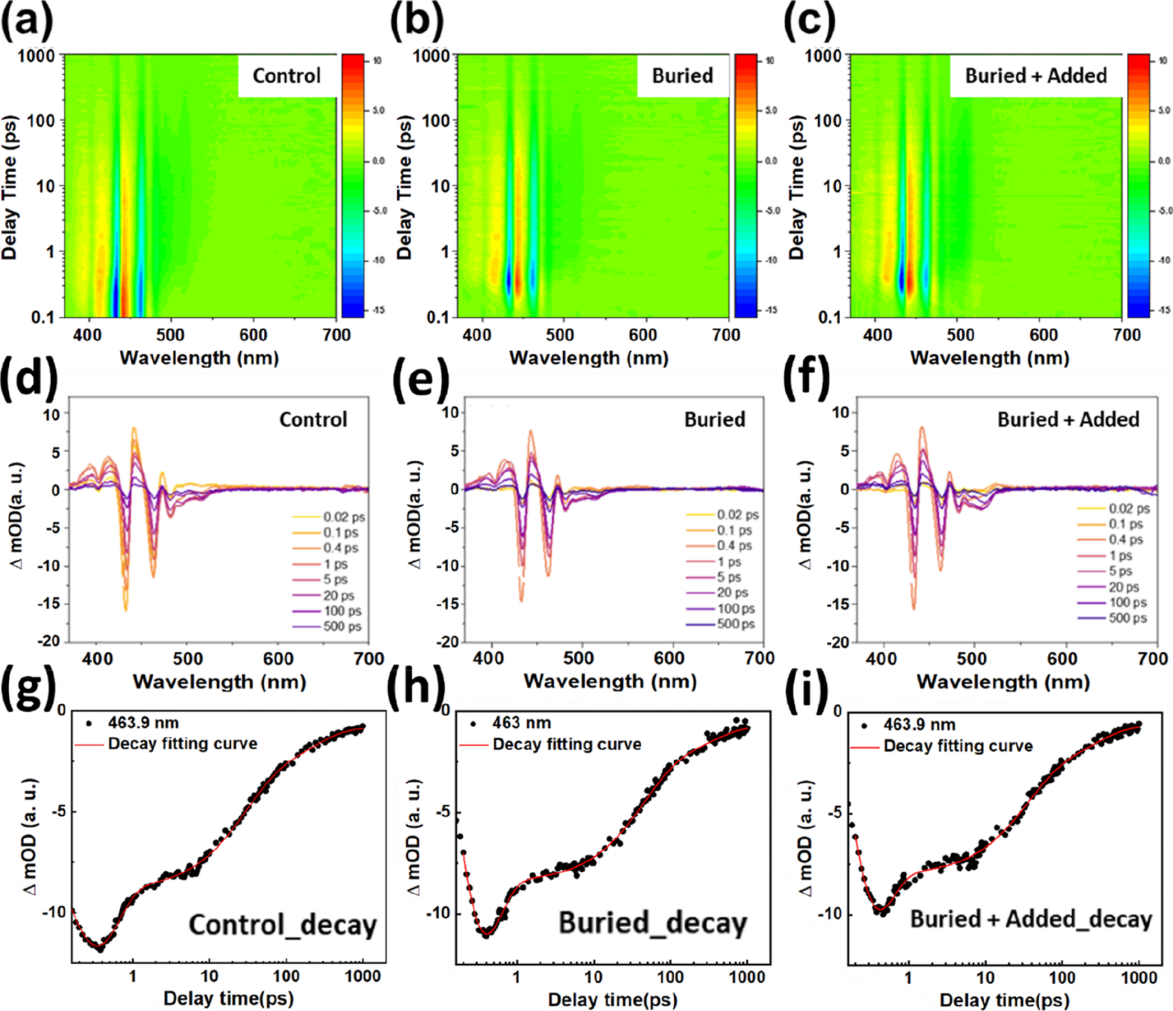
Contour plots of transient absorption spectroscopy (TAS) for perovskite films with different GBAC treatments: (a) control, (b) buried and (c) buried + added. Time-dependent absorption spectra of perovskite films at different stages of carrier dynamics under different GBAC treatments: (d) control, (e) buried and (f) buried + added. TAS decay fitting at 464 nm (*n* = 3 phase) for different perovskite films: (g) control, (h) buried, and (i) buried + added. Experimental data are shown in black, and the fitted curves are presented in red.

To further dissect the energy transfer process, Figure S4 extracts selected wavelength kinetic traces for the *n* = 2 (∼434 nm), *n* = 3 (∼464 nm), and *n* ≥ 4 (∼482 nm) domains. These traces reveal rapid decay in the low-*n* phases, while the high-*n* phases exhibit slower rise and decay rates, confirming the funnel-like energy transfer mechanism from the low-*n* to high-*n* domains. For the time traces extracted at 464 nm (*n* = 3) peak, a triexponential function incorporating an energy transfer term was employed for fitting by eq 1

1 $$\Delta A\;\left(t\right)=a_{1}e^{-t/\tau 1}+a_{2}e^{-t/\tau 2}+a_{3}e^{-t/\tau 3}-ce^{-t/\tau et}$$

where τ_et_ represents the formation (rise) time of the *n* = 3 signal driven by energy transfer from the lower-*n* domains (inflow). In contrast, τ_1_–τ_3_ describes the subsequent decay/relaxation dynamics of the *n* = 3 population, including: ultrafast interdomain transfer/outflow processes to higher-*n* emissive convergence domains (τ_1_), intermediate relaxation processes potentially involving trap-assisted nonradiative pathways (τ_2_), and long-lived bleach recovery/recombination dynamics (τ_3_).^34,40^ Figure 5g–i and Table S2 summarize the fitting results. In the control sample, τ_1_ = 0.22 ps and τ_et_ = 0.22 ps, whereas these values decreased to 0.18 and 0.18 ps in the buried condition. Under the buried + added condition, they further decreased to 0.17 and 0.17 ps. The synchronous reduction of τ_1_ and τ_et_ indicates enhanced interdomain carrier transfer efficiency and narrower phase distribution, consistent with our previous findings. These findings highlight GBAC’s pivotal role in promoting interdomain energy transfer (exciton/energy funneling effect) and optimizing quasi-2D phase organization. Such transfer-accelerating dynamics align with the quasi-2D energy cascade (MQW) model reported in the literature—by regulating phase distribution and interdomain coupling, the funnel transport pathway from low-*n* domains to high-*n* domains can be smoothed.^33^

To evaluate the practical impact of our dual-functional guanidinium-based engineering strategy, we fabricated sky-blue PeLEDs with the structure shown in Figure 6a: ITO/NiO_*x*_/Br-2PACz/PVK/GBAC-modified perovskite (with or without GBAC additive)/TPBi/LiF/Al, with fabrication details provided in the Experimental Section. The energy level diagram obtained via ultraviolet photoelectron spectroscopy (UPS) (Figures S5–S7) reveals a significant energy barrier of ∼0.30 eV between the highest occupied molecular orbital (HOMO, −5.80 eV) of PVK and the valence band maximum (VBM, −6.10 eV) of the perovskite layer in the control device (Figure 6b, left). This barrier hinders hole injection and leads to charge imbalance. Introducing a buried GBAC interlayer effectively deepens the PVK’s HOMO level to −6.04 eV by adjusting its interfacial dipole moment, thereby reducing energy mismatch at the HTL/emissive layer interface (Figure 6b, right). Simultaneously, direct addition of GBAC into the perovskite precursor slightly shifts the perovskite’s HOMO level upward to −5.98 eV (Figure 6b, right). This shift originates from the chemical coordination between GBAC and Pb^2+^ ions, as well as its interaction with organic spacers, thereby tuning the perovskite’s electronic structure.^41^ Collectively, these two effects reduce the energy barrier to a negligible level (∼0.06 eV), achieving optimal energy-level alignment under the buried + added condition.

**Figure 6:**
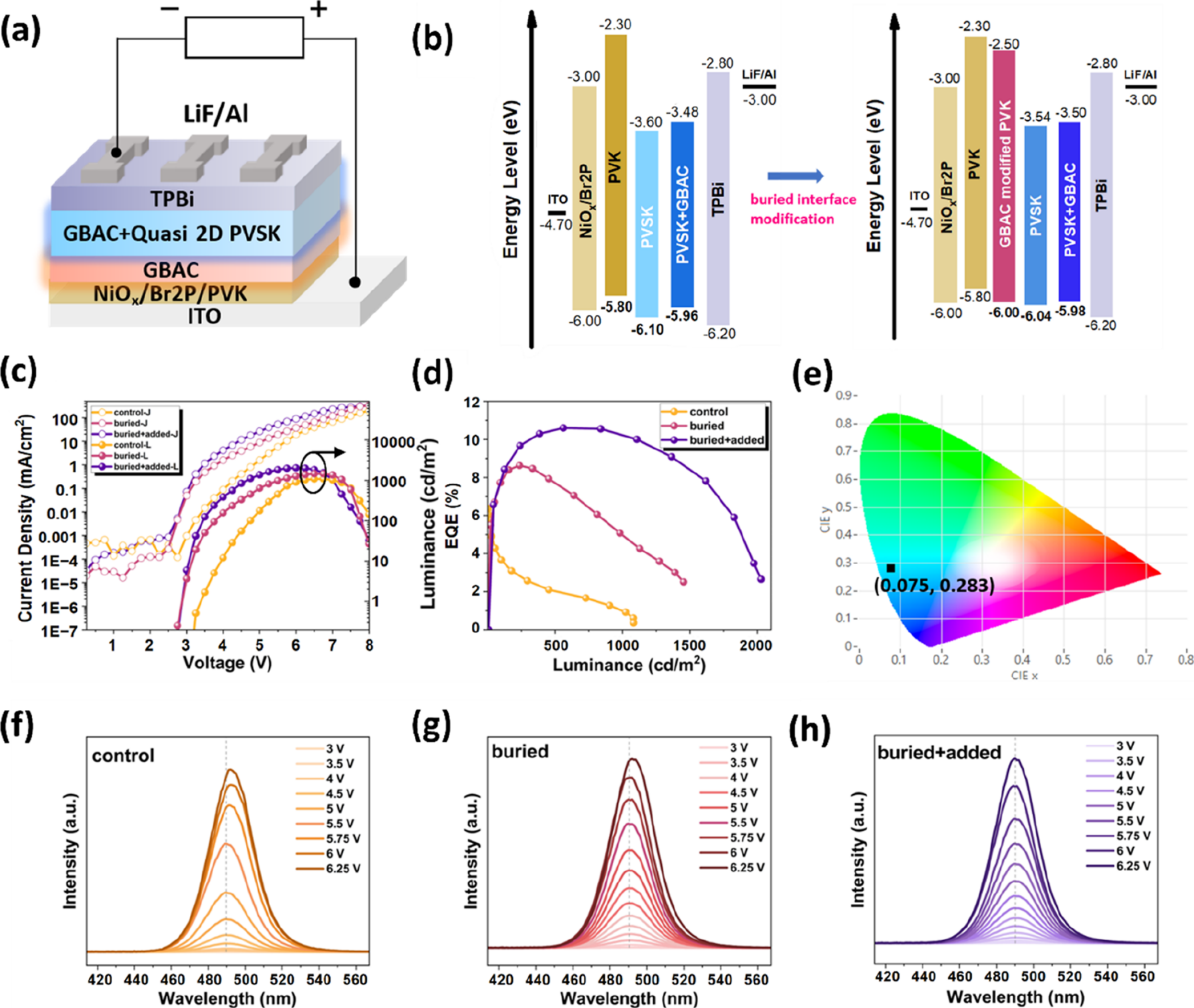
(a) Structure of PeLED. (b) Energy level diagram of the devices with and without GBAC interface treatment. (c) Current density–voltage–luminance (*J*–*V*–*L*) curves and (d) EQE-luminance curves. (e) Commission Internationale de l’Eclairage (CIE) coordinate and (f–h) EL spectra of control, buried and buried + added devices.

This optimized alignment promotes more efficient hole injection and transport, suppresses charge accumulation, and enhances device performance in current density–voltage–luminance (*J–V–L*) curves and EQE metrics. Device performance metrics are summarized in Table S3. The control device exhibits a maximum EQE of 6.37% and peak luminance of 1082.2 cd/m^2^. Upon introducing GBAC at the buried interface, EQE increased to 8.63% with luminance reaching 1453.2 cd/m^2^, indicating effective suppression of nonradiative recombination. When GBAC was simultaneously incorporated as both a buried interlayer and a bulk additive, performance further improved, achieving a peak EQE of 10.60% and maximum luminance of 2026.7 cd/m^2^. Notably, all devices exhibited an electroluminescence (EL) peak at 489 nm (sky-blue emission), indicating consistent color purity across different treatments. The constant EL peak position is consistent with PL analysis results (Figure 3c), indicating that GBAC primarily optimize the phase structure and promotes interdomain transfer toward the same emissive convergence domains, rather than transforming the dominant emissive phase into a lower-bandgap component.^33^ As further demonstrated by the *J–V–L* characteristics (Figure 6c), the buried + added device outperforms other device types s in both current efficiency and luminance. The turn-on voltage of the control device is 3.0 V, whereas both buried and buried + added devices exhibit reduced turn-on voltages down to 2.75 V. This reduction in drive threshold indicates enhanced charge injection efficiency, attributed to improved energy level alignment at the perovskite/HTL interface and within the emissive layer, as verified by prior UPS results.

Additionally, GBAC-treated devices exhibited significant leakage current attenuation in the voltage range below the EL threshold. The control device exhibits a distinct leakage current tail near the turn-on point, indicating charge loss due to interfacial defects and traps. In contrast, both buried and buried + added devices display leakage current suppression, confirming GBAC’s role in defect passivation and film densification. This suppression of nonradiative leakage pathways further corroborates the observed improvements in EQE and operational stability. As shown in Figure 6d, the EQE-luminance curve remains stable and exhibits higher values across the entire luminance range, reflecting superior radiative efficiency and effective suppression of exciton quenching under high injection conditions. The EQE–current density and EQE–voltage curves are detailed in Figure S8, showing consistent trends. Furthermore, the device’s Commission Internationale de l’Eclairage (CIE) coordinate (Figure 6e) reaches (0.075, 0.283), approaching the Rec. 2020 standard for blue display applications. Voltage-dependent EL spectra (Figure 6f–h) reveal a wavelength red shift in the control device at high voltages, indicating phase instability and ion migration. In contrast, both the buried and buried + added devices maintain spectral stability with only negligible shifts, highlighting GBAC’s effectiveness in suppressing phase redistribution and stabilizing the emission zone under voltage stress.

To further investigate the influence of GBAC on charge transport characteristics and defect distribution within the perovskite emissive layer, space-charge-limited current (SCLC), electrochemical impedance spectroscopy (EIS), and capacitance–voltage (*C*–*V*) measurements were performed on hole-only or complete PeLED device structures under dark conditions. To quantify the trap density (*N*_t_) within the perovskite emissive layer, SCLC measurements were performed using hole-only devices with a device configuration of ITO/NiO_*x*_/Br-2PACz/PVK/(GBAC)/perovskite with or without GBAC/MoO_3_/Ag. As shown in Figure 7a–c, the current–voltage (*J–V*) characteristics in SCLC exhibit three distinct regions: (i) an ohmic region at low bias (*V* < trap-filled limit voltage (*V*_TFL_)), where current increases linearly with voltage, primarily driven by thermally excited free carriers; (ii) The trap-filled limit (TFL) region, characterized by a sharp current increase when *V*_TFL_ is reached. This transition point indicates complete filling of all trap states in the emissive layer, with subsequent injected carriers directly contributing to space charge; (iii) the child region (also termed the trap-free SCLC region) at high bias, where current exhibits quadratic dependence on voltage (*J* ∝ *V* ^*2*^) – a hallmark of space-charge-limited conduction unaffected by traps. Through SCLC analysis, VTFL can be derived. This parameter is directly related to *N*_t_ in the active layer, with the relationship expressed in eq 2

2 $$N_{t}=\frac{{2\varepsilon \varepsilon_{0}V}_{TFL}}{eL^{2}}$$

where ε is the dielectric constant of perovskite, ε_0_ is the vacuum permittivity, *e* is the elementary charge, and *L* is the active layer thickness. As can be seen, the control device exhibits a relatively high *V*_TFL_ of 0.55 V, corresponding to a higher *N*_t_. Upon introducing GBAC at the buried interface, *V*_TFL_ decreases to 0.47 V, indicating reduced trap density. Under the buried + added condition, a further reduction to 0.43 V was observed, confirming GBAC’s effectiveness in suppressing trap densities within the emissive layer.^34^ This result strongly correlates with the decrease in nonradiative rate constant (*k*_nonrad_) and improved TRPL lifetime, further validating GBAC’s effectiveness in mitigating nonradiative losses.

**Figure 7:**
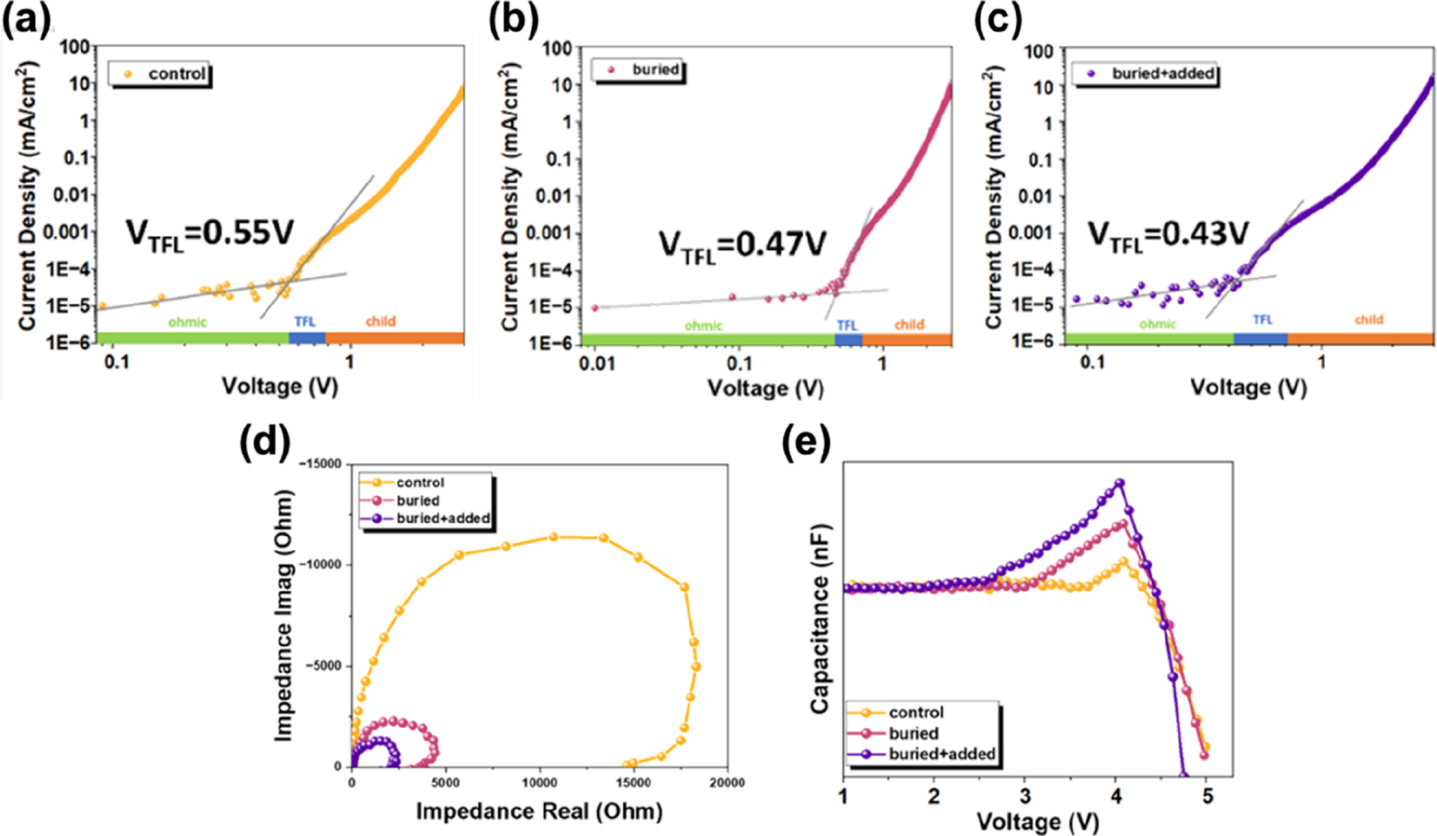
SCLC measurement results for hole-only devices under (a) control, (b) buried, and (c) buried + added conditions. (d) Nyquist plots from EIS analysis of control and GBAC-treated devices. (e) *C*–*V* curves for control and GBAC-treated devices.

EIS was then employed to investigate the charge transport characteristics and interfacial behavior of the devices. In the Nyquist plots (Figure 7d), all samples exhibited characteristic semicircles in the high-to-medium frequency range. This corresponds to the charge transfer resistance (*R*_ct_) at the perovskite/charge transport layer interface and the recombination dynamics within the emissive layer.^42,43^ A larger semicircle indicates a higher interfacial resistance and slower charge transfer kinetics, while a smaller semicircle reflects lower resistance and more efficient charge transport at the device interface. As observed, the control device exhibits the largest semicircle, indicating poor interfacial charge transfer and higher recombination losses. Upon introducing GBAC as the buried interfacial layer, the semicircle diameter significantly decreased, with further reduction observed in the buried + added device. This progressive reduction indicates that GBAC treatment effectively improves interfacial contact, enhances crystallinity, and reduces defect density. These combined effects facilitate more efficient carrier extraction and fewer recombination pathways.^44–47^ These findings align with the improved *J–V–L* characteristics, confirming GBAC’s dual role in passivating interfacial traps and optimizing charge injection balance. To evaluate the significance of our device performance, a comparison with representative sky-blue/blue PeLEDs reported in the literature is presented in Table S4.^48–51^

Finally, *C*–*V* measurements were measured to probe the charge accumulation dynamics and injection balance under forward bias (Figure 7e). In a typical *C*–*V* curve for a PeLED, the initial rising segment at low bias reflects charge accumulation within the device—formed by carrier injection into the emissive layer followed by storage at interfaces or trap states. A steeper slope in this region indicates higher carrier injection efficiency and greater charge storage capacity. Our results show that GBAC-treated devices (both buried and buried + added) exhibit a more pronounced capacitance rise compared to the control device, indicating enhanced carrier injection and accumulation efficiency due to improved interfacial energy states and reduced injection barriers. The subsequent capacitance drop observed in the high-voltage region corresponds to the state where injected carriers undergo radiative or nonradiative recombination. The buried + added device exhibited a faster capacitance decay rate, indicating more balanced carrier injection and higher recombination efficiency, suggesting fewer residual carriers within the device. These improvements correlate with enhanced EL performance, highlighting the effectiveness of GBAC in modulating the electronic landscape of quasi-2D perovskite films.^34^

## Conclusion

We have successfully validated an efficient dual-function strategy using GBAC as both a buried interface modifier and an additive to enhance the performance of all-bromide quasi-2D sky-blue PeLEDs. Introducing GBAC at the buried interface significantly improved film wettability and energy alignment within the device, while its use as an additive facilitated phase distribution control and enhanced crystallinity. Given these improvements, the device employing the buried interface modification plus additive strategy achieved over 60% performance enhancement compared to the pristine device, with a maximum EQE of 10.6%. Concurrently, both brightness and stability were significantly enhanced. Comprehensive optical, morphological, and electrical analyses reveal the synergistic mechanism by GBAC coregulates perovskite crystallization and interfacial energetics. This study provides a simple and effective engineering strategy for developing spectrally pure, stable, and highly efficient all-bromide PeLEDs.

## Supplementary Material



## Notes

Which includes detailed materials and methods, additional GIWAXS patterns and linecuts, TRPL fitting parameters with extracted *k*_rad_/*k*_nonrad_, XPS spectra (Br 3d and N 1s), TAS kinetic traces with fitting results, UPS spectra for energy-level determination, as well as additional device performance data (EQE–*J*, EQE–*V*) and comparison tables (PDF)
