# Supplementary material for: Enhancing Sky-Blue Perovskite Light-Emitting Diode Performance through Guanidinium-Based Dual-Functional Molecular Engineering
Source: ACS Appl Mater Interfaces. 2026 Jan 22;18(4):7189–201. doi: 10.1021/acsami.5c23193 (PMC12884467; doi:10.1021/acsami.5c23193)
Supplement: Supplementary file 1 [file am5c23193_si_001.pdf]

# Supporting Information

# Enhancing Sky-Blue Perovskite Light-Emitting Diode Performance through Guanidinium-Based Dual-Functional Molecular Engineering

*Yu-Hsiang Teng,[a] Hou Li,[a] Chiung-Han Chen,[a] Yen-Yu Wang,[b] Bi-Hsuan Lin,[c] I-Chih Ni,[d] Chi-Ching Kuo,[e] Yu-Jung Lu,[b] and Chu-Chen Chueh[a]**

[a] Department of Chemical Engineering, National Taiwan University, Taipei 10617, Taiwan

[b] Research Center for Applied Sciences Academia Sinica Taipei 11529, Taiwan

[c] National Synchrotron Radiation Research Center, Hsinchu 30076, Taiwan

[d] Graduate Institute of Photonics and Optoelectronics, National Taiwan University, Taipei 10617, Taiwan

[e] Department of Molecular Science and Engineering, Institute of Organic and Polymeric Materials, National Taipei University of Technology, Taipei 106, Taiwan

* Corresponding author. E-mail: cchueh@ntu.edu.tw

**Keywords:** Perovskite light-emitting diodes; sky-blue emission; dual-functional molecular engineering; defect passivation; phase distribution regulation

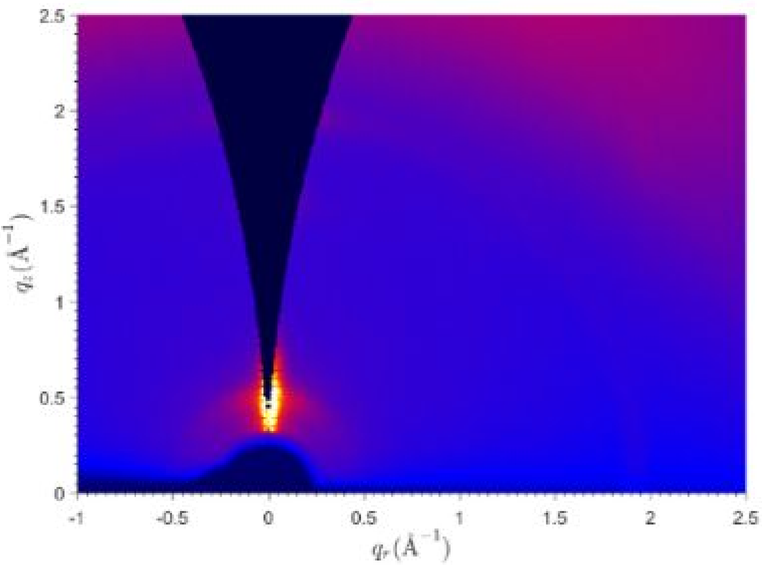


**Figure S1.** GIWAXS pattern of the perovskite film with GBAC at buried interface.

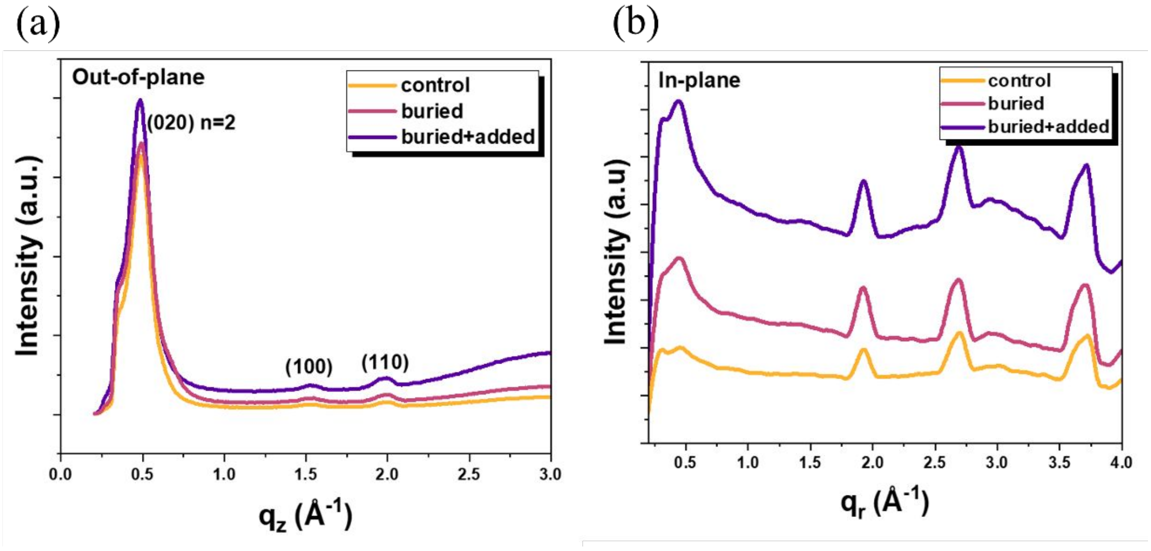


**Figure S2.** (a) Out-of-plane and (b) In-plane GIWAXS analysis of control, buried, and buried + add perovskite films.

**Table S1.** TRPL fitting parameters and radiative and non-radiative recombination rates of perovskite films without and with different GBAC treatments.

| | $A_1$ | $\tau_1$ | $A_2$ | $\tau_2$ | $\tau_{avg}$ (ns) | $k_{rad}$ (x $10^7$ $s^{-1}$) | $k_{nonrad}$ (x $10^7$ $s^{-1}$) |
|---|---|---|---|---|---|---|---|
| **control** | 0.57 | 1.44 | 0.43 | 3.70 | 2.40 | 6.82 | 34.7 |
| **buried** | 0.72 | 1.78 | 0.33 | 4.63 | 2.68 | 8.52 | 28.8 |
| **buried + added** | 0.84 | 2.10 | 0.21 | 5.87 | 2.87 | 10.2 | 24.6 |

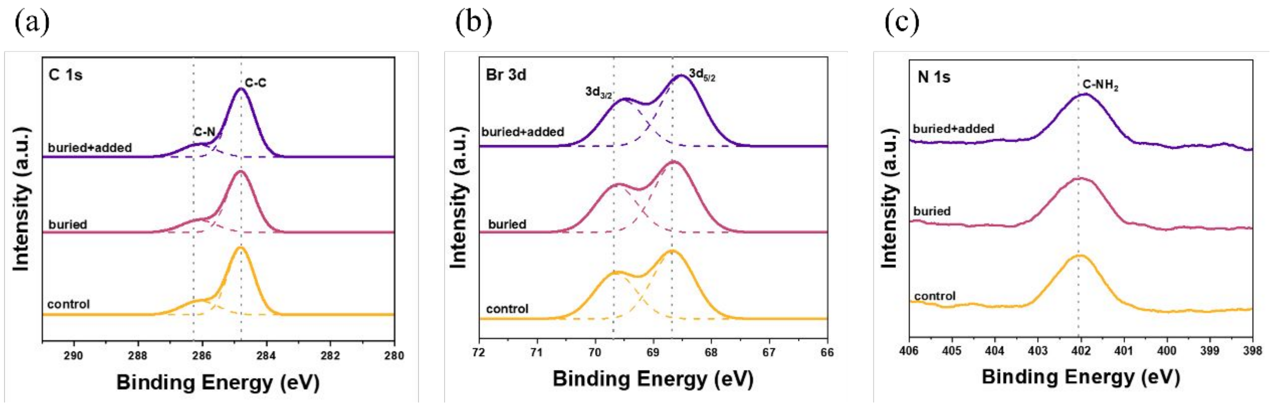


**Figure S3.** Core-level XPS spectra for (a) C 1s, (b) Br 3d, and (c) N1s for control, buried, and buried + added conditions.

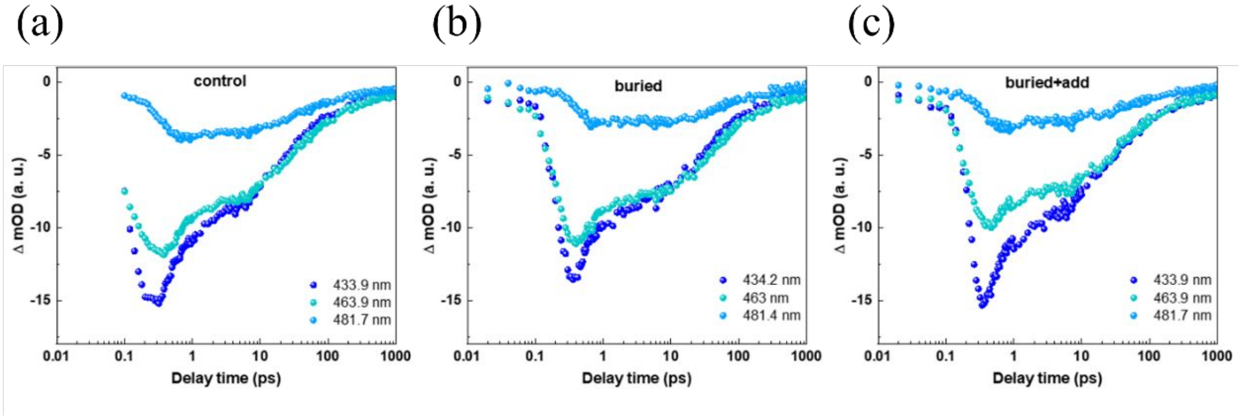


**Figure S4.** Kinetic decay profiles extracted from characteristic wavelengths in transient absorption spectra: (a) control, (b) buried, and (c) buried + added perovskite films.

**Table S2.** Fitting parameters extracted from TAS decay curves at 464 nm ($n$ = 3 phase) for control, buried, and buried + add films using a tri-exponential plus energy-transfer model.

| | $\tau_1$ | $\tau_2$ | $\tau_3$ | $\tau_{et}$ |
|---|---|---|---|---|
| **control** | 0.22 | 27.67 | 217.58 | 0.22 |
| **buried** | 0.18 | 42.20 | 318.00 | 0.18 |
| **buried + added** | 0.17 | 32.71 | 255.71 | 0.17 |

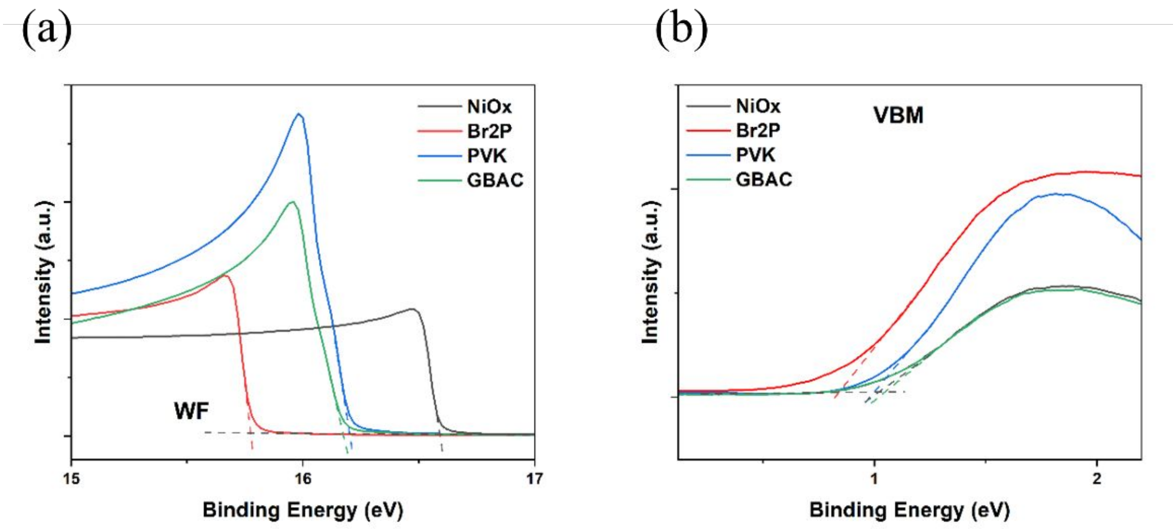


**Figure S5.** (a) Secondary electron cutoff region used for work function (WF) determination; (b) Valence band edge region used for extracting the valence band maximum (VBM) of the underlying transport and interface layers (ITO/$NiO_x$; ITO/$NiO_x$/Br-2PACz; ITO/$NiO_x$/Br-2PACz/PVK; and ITO/$NiO_x$/Br-2PACz/PVK/GBAC).

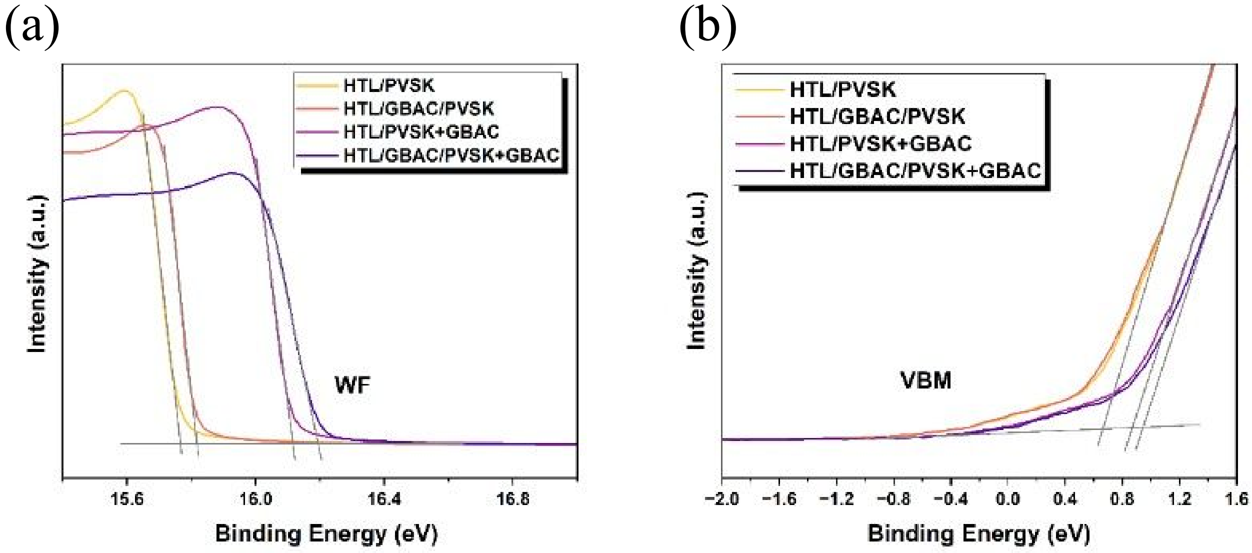


**Figure S6.** (a) Secondary electron cutoff region used for WF determination; (b) Valence band edge region used for extracting the VBM for perovskite films subjected to different GBAC treatments, corresponding to ITO/$NiO_x$/Br-2PACz/PVK/PVSK; ITO/$NiO_x$/Br-2PACz/PVK/GBAC/PVSK; ITO/$NiO_x$/Br-2PACz/PVK/PVSK+GBAC; and ITO/$NiO_x$/Br-2PACz/PVK/GBAC/PVSK+GBAC.

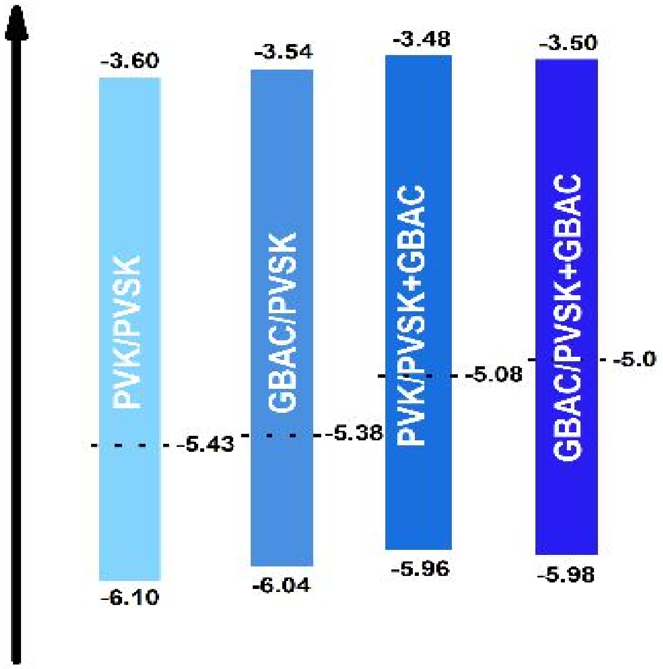


**Figure S7.** Energy level diagrams of perovskite films with different GBAC treatments, constructed from the WF and VBM extracted from **Figures S5** and **S6**, showing the Fermi level alignment states.

**Table S3.** Performance of fabricated PeLEDs.

| | $V_{on}$ (V) | EL peak (nm) | EQE (%) | Max. Lum. (cd/m$^2$) |
|---|---|---|---|---|
| **control** | 3 | 489 | 6.37 | 1082.2 |
| **buried** | 2.75 | 489 | 8.63 | 1453.2 |
| **buried + added** | 2.75 | 489 | 10.60 | 2026.7 |

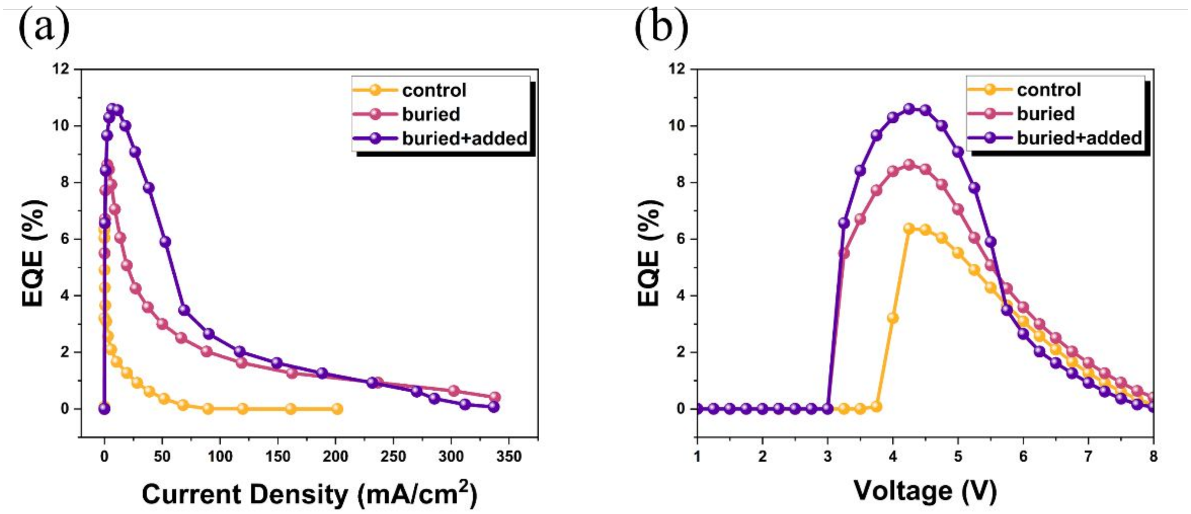


**Figure S8.** (a) EQE–current density and (b) EQE–voltage characteristics for PeLEDs fabricated under three different conditions: control, buried, and buried + added.

**Table S4.** Literature comparison of representative sky-blue/blue PeLEDs.

| Year | EL (nm) | EQE (%) | Max. Lum. (cd/m$^2$) | Ref. |
|---|---|---|---|---|
| ***2024*** | 488 | 8.70 | 642 | **[48]** |
| ***2024*** | 484 | 7.82 | 5127 | **[49]** |
| ***2025*** | 490 | 8.70 | 2480 | **[50]** |
| ***2025*** | 488 | 9.56 | 3321 | **[51]** |
| ***2025*** | 489 | 10.60 | 2026.7 | **This work** |
